# European land CO_2_ sink influenced by NAO and East-Atlantic Pattern coupling

**DOI:** 10.1038/ncomms10315

**Published:** 2016-01-18

**Authors:** Ana Bastos, Ivan A. Janssens, Célia M. Gouveia, Ricardo M. Trigo, Philippe Ciais, Frédéric Chevallier, Josep Peñuelas, Christian Rödenbeck, Shilong Piao, Pierre Friedlingstein, Steven W. Running

**Affiliations:** 1 Laboratoire des Sciences du Climat et de l'Environnement, LSCE/IPSL, CEA-CNRS-UVSQ, Université Paris-Saclay, F-91191 Gif-sur-Yvette, France; 2 Instituto Dom Luiz, IDL, Faculdade de Ciências, Universidade de Lisboa, Lisboa 1749-016, Portugal; 3 Department of Biology, University of Antwerp, Universiteitsplein 1, 2610 Wilrijk, Belgium; 4 CREAF, Cerdanyola del Vallès, Catalonia, 08193 Barcelona, Spain; 5 CSIC, Global Ecology Unit CREAF-CSIC-UAB, Cerdanyola del Vallès, Catalonia, 08193 Barcelona, Spain; 6 Max Planck Institute for Biogeochemistry, Jena 07701, Germany; 7 Department of Ecology, College of Urban and Environmental Sciences, Peking University 5 Yiheyuan Road, Haidian District, Beijing 100871, China; 8 College of Engineering, Mathematics and Physical Sciences, University of Exeter, Exeter EX4 4QF, UK; 9 Numerical Terradynamic Simulation Group, University of Montana, Missoula, Montana 59812, USA

## Abstract

Large-scale climate patterns control variability in the global carbon sink. In Europe, the North-Atlantic Oscillation (NAO) influences vegetation activity, however the East-Atlantic (EA) pattern is known to modulate NAO strength and location. Using observation-driven and modelled data sets, we show that multi-annual variability patterns of European Net Biome Productivity (NBP) are linked to anomalies in heat and water transport controlled by the NAO–EA interplay. Enhanced NBP occurs when NAO and EA are both in negative phase, associated with cool summers with wet soils which enhance photosynthesis. During anti-phase periods, NBP is reduced through distinct impacts of climate anomalies in photosynthesis and respiration. The predominance of anti-phase years in the early 2000s may explain the European-wide reduction of carbon uptake during this period, reported in previous studies. Results show that improving the capability of simulating atmospheric circulation patterns may better constrain regional carbon sink variability in coupled carbon-climate models.

## Abstract (web summary)

The relationship between terrestrial carbon sinks and atmospheric modes of variability remains uncertain. Here, the authors show that the coupling of the North Atlantic Oscillation and East-Atlantic patterns explains variations in the European CO_2_ sink from 1982 to 2012.

## Graphical abstract

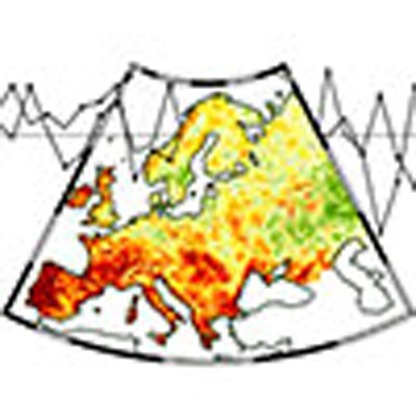

Fundamental patterns of atmosphere-ocean variability are explained by teleconnections at the global^1^ and regional^2^,^3^,^4^ scales. At the global scale, the El-Niño/Southern-Oscillation has been shown to influence inter-annual variability in the global land carbon sink^5^,^6^ with an evident fingerprint in atmospheric CO_2_ growth rate fluctuations^7^, highlighting their impact on ecosystem functioning. Hallet *et al*.^8^ proposed that teleconnections explain ecological processes even better than single climate variables, because they influence simultaneously the range of weather variables that elicit interacting, and sometimes opposing, responses by ecosystems^9^,^10^,^11^,^12^.

European and North-American climate is particularly influenced by the North-Atlantic Oscillation (NAO), which corresponds to a meridional dipole of sea-level pressure (SLP) variability between Iceland and the Azores that controls the location and strength of storm tracks in the North-Atlantic^3^ (Fig. 1a). NAO drives patterns and extremes in temperature, precipitation, snow cover and wind in Europe, especially during winter^2^,^4^, although its effects may propagate through the following seasons^13^. NAO is usually associated with warmer and wetter (colder and drier) winters in northern (southern) Europe during positive phase and approximately the reverse pattern in negative phase^2^. Some studies have analysed impacts of NAO on European ecosystems^14^,^15^. In the Iberian Peninsula, Carnicer *et al*.^16^ found an increase in tree defoliation in response to a period of drought (1990–1995) that coincided with the persistence of positive NAO. However, other teleconnections are known to also influence the European climate, particularly the East-Atlantic (EA) pattern that has a similar configuration as NAO, albeit displaced southwards (Fig. 1a). Although EA and NAO indices are computed as independent variables (Fig. 1b), the EA pattern has been proposed to modulate the multi-decadal variability of the location and strength of the NAO dipole^17^. Comas-Bru and McDermott^4^ showed that the combined analysis of NAO and EA more appropriately describes winter climate variability in Europe.

Northern Hemisphere ecosystems are important carbon sinks^18^,^19^ that partially offset anthropogenic CO_2_ emissions^20^. The inter-annual variability in the terrestrial carbon sink is poorly understood^21^, hindering better future land-sink strength projections^7^,^22^ and making regional trends in sinks difficult to detect and hence controversial^23^,^24^. Various estimates of European Net Biome Productivity (NBP) have shown that European ecosystems have been a CO_2_ sink during the past decades (1980–2005)^25^,^26^, although signs of saturation in temperate forest carbon uptake in the early 2000s have been reported^27^. Moreover, Piao *et al*.^28^ have found a weakening relationship between temperature and growing season vegetation greenness in Northern ecosystems, especially in Europe, during 1982–2011, attributable to an increase in drought conditions. Their results highlight the importance of evaluating inter-annual variability of NBP within a framework that takes into account the co-variations of the climate variables that drive ecosystem dynamics.

Here we analyse the combined impact of NAO and EA on NBP over the European area extending to the Ural Mountains (Supplementary Fig. 1), using three state-of-the-art atmospheric CO_2_ inversions: two versions of the Monitoring Atmospheric Composition and Climate—Interim Implementation (MACCII) inversion^29^,^30^ and the Jena s81 v3.6 inversion^31^ (Supplementary Table 1). Atmospheric inversions are known to provide consistent estimates of inter-annual variability of the land sink on large aggregated regions^19^. The uncertainty of the annual anomalies is expected to be lower than that of the long-term mean, since these errors on annual fluxes are positively correlated from 1 year to the next, and Europe is one of the regions with most robust inter-annual variability estimates^32^. As inversions present increasing uncertainty for smaller scale regions^33^ we use a complementary bottom-up estimation of NBP from eleven Dynamic Global Vegetation Models (DGVMs)^20^ (Supplementary Table 2). The DGVMs were forced with climate observations and include land-use changes (LUCs), but differ on a number of characteristics, which makes them independent estimations of NBP. These data sets now report more than 30 years of CO_2_ flux variability, a timely opportunity to revisit the relationship between carbon fluxes and climate signals. Our results show that the combined NAO–EA variability explains variations in the European CO_2_ sink from 1982 to 2012, through their control on heat and water-vapour transport towards the continent and consequent climate anomalies. We find that the European sink is enhanced only when the two modes are in negative phase due to enhanced photosynthesis, while for both anti-phase combinations NBP is kept below average through different responses of photosynthesis and respiration.

## Results

### Net biome production variability

**Figure 2:**
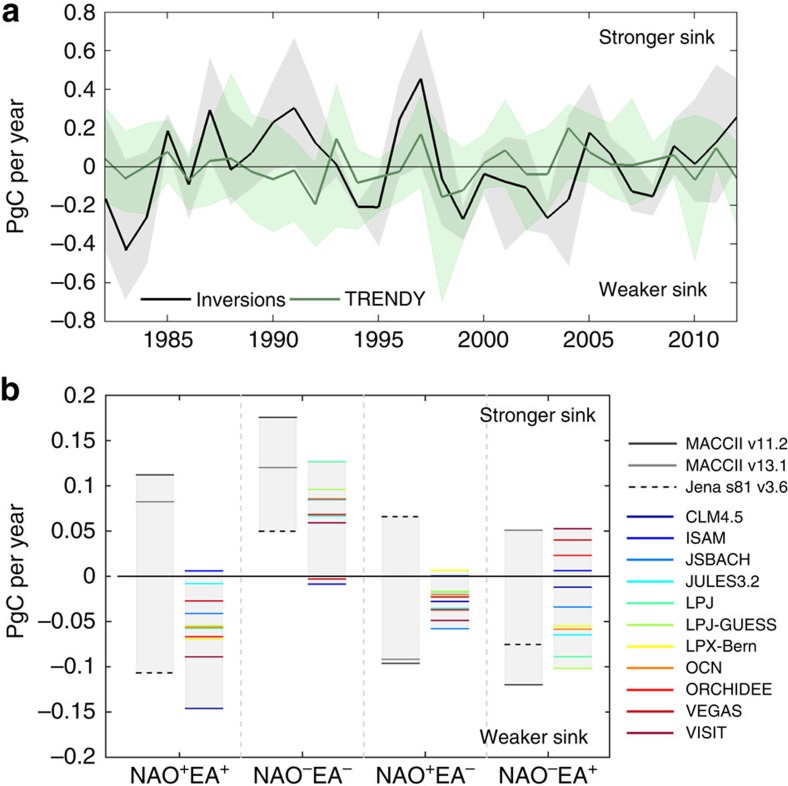
Impact of NAO–EA variability on NBP. (**a**) Annual NBP anomalies integrated over Europe (bold lines) and corresponding spread, that is, the minimum and maximum NBP estimated by each set (shaded area), for the atmospheric transport model inversions (black) and the DGVMs from TRENDY project (green). (**b**) Average European NBP anomalies for the four NAO–EA composites (Supplementary Table 3), assessed by inversions (greyscale lines) and DGVMs (colour lines).

European NBP from inversions confirms that European ecosystems were on average a net CO_2_ sink between 1982 and 2012 (Supplementary Fig. 2), in line with previous estimates^19^,^25^, with strong inter-annual and multi-year variability (Fig. 2a). The late 1980s and early 1990s were characterized by generally positive NBP anomalies, that is, CO_2_ uptake above average. A strong sink was observed during the years 1996–1997, followed by a decade of mostly below-average NBP, consistent with the satellite observations of Normalized Difference Vegetation Index (NDVI) data, a proxy for Gross Primary Productivity (GPP)^27^. From 2009 onwards, results from inversions suggest that European ecosystems became a stronger C sink again. The set of DGVMs suggests a weaker and less-variable European sink than the inversions (Supplementary Fig. 2), although the average NBP anomalies from these models remain in most years within the uncertainty range of the inversions and also present no significant trend (Fig. 2a).

Despite the lack of long-term NBP trend at continental scale, Supplementary Fig. 3 shows distinct temporal patterns in annual NBP anomalies in the four large regions encompassing most of continental Europe. The continental integral is mainly influenced by the dynamics in central Europe (33% and 34% of variance explained on average for inversions and DGVMs, respectively) and western Russia (29%, inversions). A principal component analysis (PCA) applied on NBP fields from inversions and DGVMs confirms the importance of central Europe and western Russia dynamics, by identifying a predominant variability pattern (explaining about 30% of spatio-temporal variance) with two centres located in these regions (Supplementary Fig. 4). Particularly, anomalies in central Europe dominate the multi-year pattern observed at the continental scale, while in western Russia NBP presents an increasing trend in the first decade followed by a peak in 1996–1997 and a stalling onwards (Supplementary Fig. 3). However, it must be kept in mind that atmospheric inversions do not perform as well at the regional scale as at the continental scale due to the limited, albeit still greater than in most regions, number of observation sites in Europe and to limitations of transport models.

### Impact of NAO and EA on NBP

A large spread in NBP response to climate is expected from the natural variability of the climate conditions (for example, NAO^+^ years differ in NAO strength and therefore in its impacts), as well as from carryover effects from previous year's climate conditions. For example, warm and moist conditions in 1 year promote organic matter decomposition, thereby increasing nutrient availability with impact on plant growth during subsequent years^12^. Because of this large spread of the NBP response to current year's climate, we here analyse the multi-year average NBP values within the four NAO–EA phase composites (NAO^+^EA^+^, NAO^−^EA^−^, NAO^+^EA^−^ and NAO^−^EA^+^), rather than individual years (Fig. 2b; Supplementary Table 3).

Our analysis reveals that the sole use of NAO does not suffice to explain NBP variability, because its impacts are strongly modulated by the sign of the EA. Inversions and DGVMs agree on significant differences in NBP composite anomalies during NAO^−^ years (Supplementary Tables 4 and 5), depending on the state of the EA. NBP is enhanced when EA is also negative, and is weaker than average when EA is positive (Fig. 2a; compare NAO^−^EA^−^ with NAO^−^EA^+^). In fact, both sets of NBP estimates identify NAO^−^EA^−^ as the single combination with consistent estimates of NBP enhancement (except two DGVMs), even for an extended record from 1950 to 2012 (Supplementary Fig. 5). During the other phase combinations, inversions and DGVMs report NBP anomalies of similar magnitude for the two anti-phase states (NAO^+^EA^−^ and NAO^−^EA^+^), which are significantly lower than for NAO^−^EA^−^. For NAO^+^EA^+^, MACCII inversions disagree with DGVMs and Jena inversion on the sign of the anomalies. However, the limited number of years (only four) in this composite highlights the need for caution in the interpretation of NBP anomalies for this combination. It is worth pointing that inversions present different sensitivities to NAO–EA variability: while the sign of NBP anomaly from the two MACCII versions tends to depend on the phase/anti-phase dichotomy, NBP anomalies from the Jena inversion appear to respond more strongly to EA phases.

### Mechanism

Having shown the need to consider both NAO and EA when assessing European CO_2_ uptake variability, we now propose the main components of the physical mechanisms associated, that is, linking variations in atmospheric circulation and climate variables to the anomalies observed in NBP for each NAO–EA phase combination.

**Figure 3:**
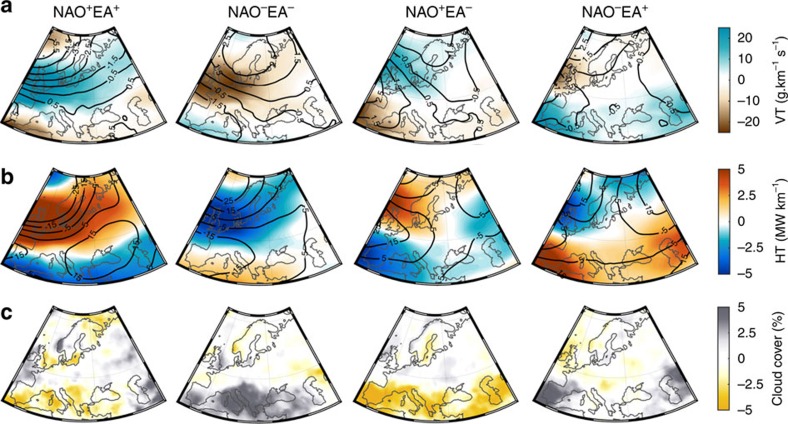
Impact of NAO–EA variability on atmospheric circulation. Anomaly fields during extended winter (Dec–Apr) for each of the four NAO–EA phases of (**a**) sea-level pressure (black contours, in mb) and eastward water-vapour transport (colours); (**b**) 500-mb geopotential height (black contours, in g.p.m.) and heat transport (colours); (**c**) Cloud cover fraction.

Variability in the land sink is linked to that in the teleconnections through the control of the latter on one or more of the meteorological variables influencing NBP. The four NAO–EA phases are characterized by distinct winter SLP anomaly patterns (December–April (Dec–Apr); Fig. 3a, black contours) and 500 mb geopotential height anomalies (Fig. 3b, black contours) during late winter/early spring. Although NAO clearly controls the sign of the North/South SLP anomaly dipole, with NAO^+^ (NAO^−^) imposing negative (positive) anomalies in the North and positive (negative) in the south, the EA modulates their strength and configuration, consistent with (ref. 4). During in-phase combinations, a sharp North/South pressure gradient is observed at the surface and in altitude, while during anti-phase combinations anomalies tend to be less intense and present a meandering configuration.

By influencing the strength and shape of the North-Atlantic jet, the interplay between NAO and EA controls the atmospheric transport of heat and humidity over the region (Fig. 3a,b; colours). The stronger pressure gradients during in-phase combinations create a channel of enhanced (weaker) eastward transport of heat and water-vapour spanning from the UK to eastern Europe and including Scandinavia, and reduced (higher) heat and moisture advection over the Mediterranean during NAO^+^ (NAO^−^). In contrast, during anti-phase periods, the weaker pressure gradients at the surface and in altitude leads to a northward displacement and attenuation of eastward heat flux, and water-vapour transport is close to average values in most of the continental Europe. The impact of these fluxes on cloud cover was evaluated as a means to estimate the combined variability of precipitation and sunlight, both of which are relevant (with opposing effects) for vegetation growth during these months^9^. The patterns of winter cloud cover anomalies roughly match the ones of heat and vapour transport, with increased cloudiness in the regions affected by stronger heat and water flux anomalies. It must be noted that although precipitation anomalies are expected to be related to cloud cover, the dependency is not direct, since precipitation also depends on vertical instability of the atmosphere.

**Figure 4:**
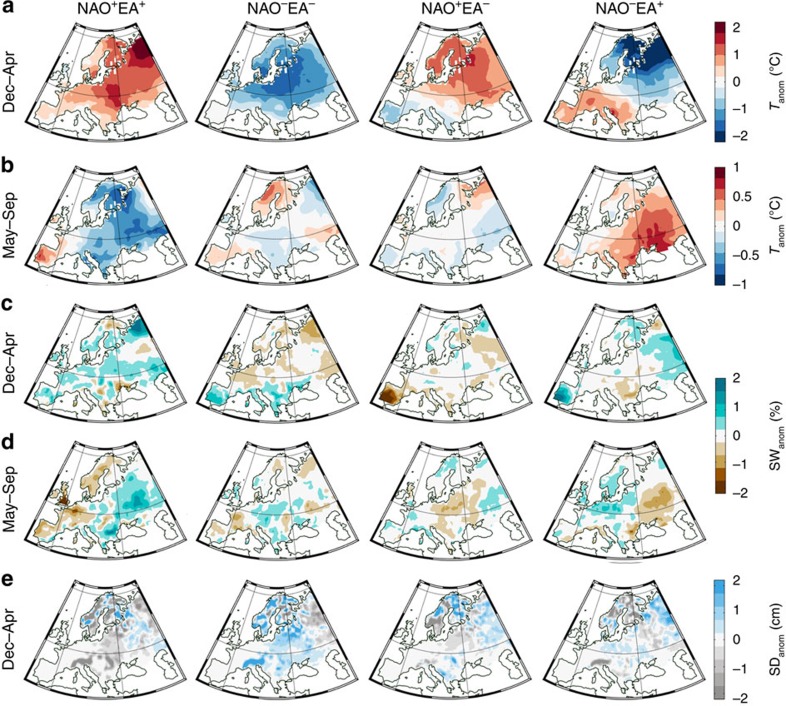
Impact of NAO–EA variability on climate variables. Average anomalies in late winter/early spring (Dec–Apr) and summer (May–Sep) of temperature (*T*, (**a**,**b**)), soil water content (SW, (**c**,**d**)) and snow depth (SD, only Dec–Apr, (**e**)) for the four NAO–EA composites.

The differences in regional advection of heat and humidity transport associated with a given phase impose distinct and spatially heterogeneous climate conditions during late winter/early spring, which may propagate until summer (May–September) due to the memory effects of snow cover^13^ and soil moisture^34^ (Fig. 4). During NAO^−^EA^−^, the inhibition of heat and water-vapour transport in winter establishes very cold conditions over most of Europe, which lead to more precipitation falling as snow rather than rain, explaining the negative soil moisture anomalies and the generally higher-than-average snow depth registered in most areas. In the Mediterranean region, soil moisture is enhanced during December–April due to the stronger advection of water vapour and prevalence of cloudiness (Fig. 3). In contrast to the lower soil moisture during winter/spring, soil water was enhanced during spring/summer in the NAO^−^EA^−^ composite in most of central Europe (Fig. 4; Supplementary Table 6). This shift in water availability may be related to later snow melt and lower evaporative losses during the colder winter/spring periods. Higher soil moisture levels support stronger latent heat exchange and thus evaporative cooling in most regions, except the Iberian Peninsula, Scandinavia and the southern section of western Russia, in which dry conditions promote higher summer temperatures. For NAO^+^EA^+^ these patterns are approximately the opposite.

The meandering SLP pattern and the corresponding energy transport during anti-phase periods impose a north-east/south-west gradient (rather than north/south as during in-phase combinations) in temperature anomalies with warmer-than-average (colder) winter/spring conditions over Scandinavia and western Russia and cooler (warmer) temperatures in the western-Mediterranean sector during NAO^+^EA^−^ (NAO^−^EA^+^). The patterns of soil moisture for NAO^+^EA^−^ (NAO^−^EA^+^) reflect the northward displacement and attenuation of water-vapour transport anomalies: most regions register close to average winter soil moisture, with drier (wetter) winter conditions being registered only in western Russia and Iberian Peninsula. Most of central Europe registers lower (higher) levels of snow cover, followed by dry (wet) soil conditions in summer. As for NAO^−^EA^−^, the snow-depth winter patterns generally match the ones observed in summer soil moisture and temperature anomalies, although the moisture–temperature coupling is most evident for NAO^−^EA^+^. The Iberian peninsula is also affected by very low winter cloud cover during NAO^+^EA^−^, which promotes higher evaporation rates, explaining the strong water deficits in winter.

**Figure 5:**
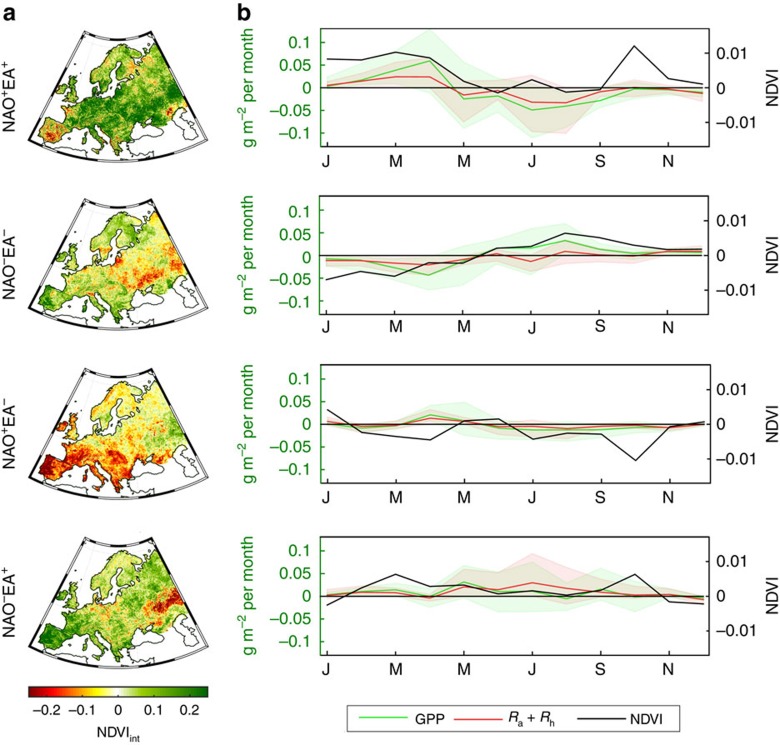
Ecological response to NAO–EA phases. (**a**) Anomaly fields of annually integrated NDVI from GIMMS for each of the four NAO–EA composites; (**b**) seasonal anomalies of NDVI (black line, right *yy*-axis), GPP (green, left *yy*-axis) and aggregated autotrophic and heterotrophic respiration (red, left *yy*-axis). For GPP and respiration, bold line corresponds to the average of the eleven DGVMs and the shaded area indicates the model spread.

The anomalies in NBP are related to two largely offsetting fluxes, photosynthesis and respiration, which respond differently to climate anomalies^12^. It is worth comparing the continental NBP response to NAO–EA phases with observations of vegetation greenness anomalies assessed by annually integrated NDVI fields (Fig. 5a) from the Global Inventory Modeling and Mapping Studies (GIMMS)^35^, as well as the corresponding seasonal evolution (Fig. 5b, black lines). Since DGVMs allow partitioning the NBP anomalies into GPP and respiration responses, the seasonal dynamics of these two components of NBP is also evaluated (Fig. 5b, coloured lines). It must be noted that since the climate anomalies are spatially heterogeneous, and Europe is composed of diverse biomes, these responses must also be assessed at the regional scale (Supplementary Figs 6 and 7, Supplementary Table 4). Regional NBP anomalies from inversions and DGVMs are compared, although inversions present higher uncertainty for smaller regions and in most cases do not present significant results. At the regional scale, DGVMs are expected to better capture ecological variability patterns in response to climate anomalies (see Methods).

Inversions and most DGVMs consistently estimate enhanced NBP during NAO^−^EA^−^ years in all regions, except in the Iberian Peninsula, where inversions and DGVMs diverge. The clear positive NBP anomaly during NAO^−^EA^−^ cannot be explained solely by positive NDVI anomalies, since large areas in Europe present NDVI anomalies of small magnitude, and in many regions even slight browning. On the seasonal scale, the cold and dry winter and spring conditions affecting most of Europe impose negative NDVI anomalies and lower-than-average GPP (especially in western Russia; Fig. 5b; Supplementary Fig. 6). High water availability due to retarded snow melt in spring and reduced evaporative soil-water loss during the colder spring sustains increased GPP from late spring to early autumn, while respiration is kept close to average values due to the mild summer temperatures, especially in parts of central Europe and western Russia. Indeed, most DGVMs estimate a strong positive dependence of summer GPP in soil water in all regions except Scandinavia (Supplementary Table 7; Supplementary Fig. 8), while only four are able to capture the known dependence of GPP in winter water availability in the Iberian Peninsula^36^. The only two DGVMs that estimate negative NBP anomalies at continental scale for NAO^−^EA^−^ (ISAM and VEGAS) present very weak control of water availability on GPP. The enhanced CO_2_ uptake during the NAO^−^EA^−^ phase combination is thus mostly due to enhanced GPP during the growing season, not to reduced respiration.

The highest positive integrated NDVI anomalies are observed for NAO^+^EA^+^ in most of Europe (except Iberia). However, the higher annual NDVI does not coincide with increased GPP, mainly because positive NDVI anomalies occur when radiation is sub-optimal (that is, late winter and early spring) and hence photosynthesis is weakly stimulated. DVGMs report higher GPP during spring, which offsets the increase in respiration during these warmer conditions. In summer, NDVI is about average, however, DGVMs estimate strong negative GPP and respiration anomalies, the former being stronger than the latter, and leading the negative NBP response (Fig. 2b). This is mainly due to the response observed in central Europe, where very dry conditions lead to very low GPP, despite the low temperatures inducing lower-than-average respiration rates (Supplementary Figs 6 and 7).

Despite both anti-phase composites resulting in negative NBP anomalies, they diverge in the corresponding climate patterns (Fig. 4). For NAO^+^EA^−^, DGVMs (inversions are consistent, but not significant) report a sink reduction in all regions except western Russia, which matches the marked vegetation ‘browning' and decrease in GPP during the year (Fig. 5) imposed by the cold and dry winter/spring in central Europe and the Mediterranean, especially in the Iberian Peninsula, and the drier summers in central Europe. This decrease in photosynthesis is accompanied by a smaller reduction in respiration, explaining the reduced NBP during this phase combination (Fig. 2; Supplementary Fig. 7). On the contrary, NAO^−^EA^+^ is characterized by overall high annual NDVI fields, associated with increased photosynthesis during spring (Fig. 5), promoted by warm and wet conditions over most of Europe, except in western Russia, where very cold late winter and spring inhibits vegetation activity (Supplementary Fig. 7; Supplementary Table 6). However, the GPP enhancement during spring is offset by greater increase in respiration during summer due to widespread positive temperature anomalies that reinforce, and are amplified by, soil dryness. This is especially evident in western Russia, where below average summer GPP is accompanied by enhanced respiration.

## Discussion

Changes in NBP are the result of variation in land cover, in land management and/or in ecosystem activity. Changes in land cover and land management are slow and not very intense in Europe, being more likely to induce long-term trends rather than short-term variation in NBP. On the inter-annual scale, NBP responds to a number of physical drivers which present co-variability patterns that are ultimately controlled by large-scale atmospheric circulation. Here, a mechanism driving part of the inter-annual and multi-year variability in the European sink is proposed, based on the interplay between the two main patterns of atmospheric circulation in the North-Atlantic sector (NAO and EA) and their control on heat and water-vapour transport across Europe.

The NBP enhancement during NAO^−^EA^−^ conditions suggests that the highest NBP (largest uptake) should occur during periods with predominantly negative phases of both teleconnections. In fact, the largest NBP peak observed in 1996–1997 in the inversions corresponds to two consecutive years of NAO^−^EA^−^. The strong increase in NBP during 1985 also occurred during this combination of NAO and EA. In contrast, the years with lowest NBP are associated with years of opposing phases of NAO and EA: the first 3 years of the period (1982–1984) and 1998–1999, when a strong decrease in NBP is observed. Anti-phase combinations dominated the late 1990s and the 2000s, with some exceptions, with a period of 6 consecutive years of anti-phase lasting from 1998 to 2003, coinciding with a weaker sink over the European continent^11^ (Fig. 2).

When computing future trends in NBP, the presence of multi-annual variability and trends in the most prominent modes of atmospheric circulation should be taken into account. In this context, anthropogenic forcing has been shown to lead to more frequent positive phases of NAO^37^, associated with lower NBP, especially when EA is negative. However CMIP3 and CMIP5 models predict opposite trends in the NAO sign over the 21*st* century under similar scenarios^38^. Shepherd^39^ pointed out that atmospheric dynamics still constitutes an important source of uncertainty in the state-of-the-art CMIP5 models, and showed that the climate-change response of the wintertime North-Atlantic jet largely differs among models, both in magnitude and sign. Other works have shown the influence of other teleconnections such as the Scandinavian Pattern^17^ or the Pacific Decadal Oscillation^40^ on North-Atlantic sea-level pressure, adding further complexity to the study of long-term NBP variability.

This study shows that the non-stationary relationship between teleconnections may strongly affect the relationships found between climate and ecosystem activity. This highlights the importance of correctly reproducing the mid-latitude atmospheric circulation patterns in Earth System Models to account for inter-annual and decadal variability in the land carbon sink. More long-term high-quality observation-driven data sets are needed to further improve our understanding of the multi-year variability of the land sink and its relation to climate variability.

## Methods

### Inversions

Atmospheric CO_2_ inversions estimate spatial distribution of surface CO_2_ fluxes using a top-down approach, in which surface fluxes are estimated from atmospheric CO_2_ concentration measurements across the globe. An atmospheric transport model that represents the global atmospheric circulation is required, as well as prior-information about the fluxes. Flux estimates over land include the carbon exchange in ecosystems, LUC related fluxes and forest fire CO_2_ emissions as well as fossil-fuel emissions. To estimate NBP, the latter are subtracted from the flux estimates.

We use monthly CO_2_ fluxes of two versions of the atmospheric inversion of the MACCII from the LSCE^29^,^30^. Version 11.2 covers the period from 1979 until 2011 and is used here from January 1982 onwards. It has a 2.5 × 3.75 (latitude, longitude) spatial resolution. Version 13.1 has a slightly higher latitudinal resolution (1.9) and covers year 2012 as well (Supplementary Table 1). Apart from the archive length, the main difference between v11.2 and v13.1 is the number of vertical layers in the underlying atmospheric transport model, the LMD General Circulation Model (LMDZ)^29^, that was doubled in the latter (39 versus 19), thus allowing a better representation of the variability of CO_2_ in the planetary boundary layer, where the assimilated measurements are located.

Since MACC inversions are based on a large but variable number of stations during the study period (134 in total), with fewer observations in the beginning of the time series, part of the variability observed on the estimated fluxes may be affected by the changes in the number of sites^41^. Thus, we use the Jena s81 (v3.6) inversion that is based on fewer stations and is provided at 4 × 5 spatial resolution (Supplementary Table 1), but uses a consistent set of sites for the 1982–2012 period. Jena inversion computes NBP on each grid cell using the atmospheric transport model TM3, as described in ref. 31.

To subtract fossil fuel emissions, MACCII inversions use the EDGAR3.2 FastTrack 2000 emission database^42^ with scaling factor to account for trends: MACC uses the annual global totals of the Carbon Dioxide Information Analysis Center and prescribes an increase of 1.4% per year after 2001. Jena inversion uses EDGAR 4.2, with FastTrack 2010 for 2009 and 2010, extrapolation based on British Petrol global totals for 2011 and 2012, and 2% year increase afterwards.

Uncertainties of annual NBP at the continental scale are given, for each model, in Supplementary Table 1. Uncertainties of the annual fluxes account for errors in the prior fluxes, network configuration (number of sites, Supplementary Fig. 1) and in the transport model. The uncertainty of the annual anomalies is expected to be lower than that of the absolute fluxes, since these errors are positively correlated from 1 year to the next^32^. We use two inversions computed with the same model but variable number of sites, and a second with a fixed number of observations, and use the spread between inversions as an indicator of the uncertainty in the anomalies.

In order to test the available signals from the smaller number of sites in Europe used by the Jena s81 inversion, we performed three synthetic-data runs (1982–2010) by transporting net ecosystem exchange (NEE) fields as simulated by the Biome-BGC DGVM in the atmosphere using the same transport model as in the Jena inversion (v3.7), though on coarser 10 × 8 spatial resolution. To create pseudo data, modelled atmospheric CO_2_ is sampled at the same locations and times as the real data for three different station sets: as in Jena s81 (14 sites) and s90 (25 sites) as well as mostly the same sites used for MACCII inversion (Supplementary Table 2). The CO_2_ concentrations sampled in this way were inverted back using the Jena scheme (setting ocean and fossil fuel priors to zero). As the same transport model is used for synthetic data and inversion, results are not contaminated by model errors. By comparing with the original fluxes (Supplementary Fig. 9), the sensitivity of the inversion skill to the site network can then be evaluated. Despite the presence of discrepancies, the run with fewer stations (Jena s81) is able to capture the variability patterns of the CO_2_ fluxes and differences in the magnitude of the fluxes are on average 0.04–0.05 PgC per year for each of the three runs.

### Dynamic global vegetation models

DGVMs simulate the main processes of vegetation dynamics and decomposition associated with energy, water and carbon balances at the ecosystem scale and provide a bottom-up approach to evaluate NBP variability, as well as the corresponding GPP and respiration components.

The TRENDY project^43^ compiles outputs from a group of DGVMs to evaluate trends and drivers in land–atmosphere carbon exchange^20^. We use 11 DGVMs (Supplementary Table 2) from simulation S3, in which all models are forced by the same values of changing atmospheric CO_2_ concentrations from ice core data and observations, historical climate observations from the CRU-NCEP v4 data set and LUC from the History Database of the Global Environment (HYDE) database of human-induced land-cover changes^44^.

Supplementary Table 2 provides a summary of the characteristics of the 11 DGVMs used in simulation S3, which is performed over the period 1860–2012. All models include deforestation, afforestation and regrowth, but differ in the way they represent disturbances (fire), nutrient limitation and do not realistically simulate land-management practices or crop seasonality, which strongly interplays with climate in determining NBP of European ecosystems. Nevertheless, DGVMs provide an independent data set to evaluate the variability of the land carbon sink. NBP from DGVMs was selected for two periods: the common period with the inversions and satellite data (1982–2012) for the main analysis, and the common period with the NAO and EA indices (1950–2012), except for JSBACH whose record ends in 2005. To partition NBP, continental and regional GPP and respiration (computed as the sum of autotrophic and heterotrophic respiration components) anomalies from the DGVMs were also evaluated between 1982 and 2012.

### NDVI

Biweekly NDVI fields over 1982–2012 from the GIMMS NDVI data set, provided at 8 km spatial resolution^35^. NDVI values were integrated over each year on a pixel basis from the biweekly fields. NDVI anomalies were then computed as the departure from the average annual integral values, and used as a proxy for GPP anomalies^28^.

Monthly NBP values from inversions and DGVMs were first deseasonalized (mean seasonal-cycle removed) on a pixel basis to compute monthly anomaly and integrated over the year for annual NBP anomaly fields, for the European continent and for the regional boxes (Supplementary Fig. 3). A positive (negative) sign of NBP anomalies correspond to either an enhanced (weaker) sink or a smaller (increased) source. To evaluate the main pattern of NBP variability, a PCA was performed on NBP fields for inversions and DGVMs. Since results from PCA depend on the resolution of the data set, all fields were resampled to a common 1° spatial resolution. The first principal component of NBP variability (PC_1_), the corresponding empirical orthogonal function (EOF_1_) and explained variance were selected for each inversion and model. EOF_1_ presented in all cases dipolar pattern. The consistency of these patterns was compared by locating the corresponding centres of action for each data set (Supplementary Fig. 4).

All climate variables used for this analysis were extracted from ERA-Interim reanalysis^45^: vertical integral of eastward transport water vapour (VT, in g km^−1^ s^−1^) and heat (HT, in MW km^−1^), fraction of cloud cover (%), SLP (in mb), 500-mb geopotential height (z500, in geopotential metres, g.p.m.), average air temperature at 2 m (*T*, in °C), volumetric soil-water content (SW, in % of volume) in the top layer^46^ and average snow depth (SD, in cm). The data comprise monthly average fields from 1982 to 2012, at 0.75 spatial resolution.

As complementary information about soil-water conditions, monthly values of the self-calibrated Palmer Drought Severity Index (PDSI)^47^, provided by the NOAA/OAR/ESRL PSD, Boulder, CO, USA at 2.5 spatial resolution was used. Since PDSI presented regional dependence (negative for Iberian Peninsula and central Europe and positive for western Russia and Scandinavia), we estimated drought conditions as the PDSI departure from the regional average (Supplementary Table 6).

### Atmospheric circulation

We focus our analysis on the impacts of winter state Dec-Feb (DJF) of two teleconnections, using the monthly indices from NOAA's Climate Prediction Centre, which are calculated by rotated PCA of monthly means of 500-mb geopotential height anomalies from NCEP/DOE II (ref. 48) as described in (ref. 49). This procedure allows the calculation of orthogonal (that is, non-correlated) indices for each month. The first mode corresponds to the NAO pattern, the leading mode of SLP variability in the North Atlantic and the main climate teleconnection affecting European weather. The second mode corresponds to EA circulation pattern. These modes affect more significantly European weather during winter^2^,^50^. For visualization of the spatial patterns associated with the two teleconnections used in this work, we performed a PCA on standardized monthly means of 500-mb geopotential height from NCEP/DOEII Reanalysis over the North-Atlantic region between 20 to 80 N and 90 W to 50 E for the winter months (DJF) as in ref. 49.

The phases of an index are defined as those exceeding the lower (negative) or upper (positive) terciles, and identified in superscript (for example, NAO^+^ for a positive phase of winter NAO). To evaluate the combined impacts of both teleconnections, we identify four subsets of years corresponding to the four possible NAO–EA phase combinations, together with a neutral composite (Supplementary Table 3).

### Regional fluxes

Since data sets are provided at very different spatial resolutions (Supplementary Tables 1 and 2), and because inversions perform better on larger scales, we consider regionally integrated NBP from inversions and DGVMs for the European continent defined in the TransCom inter-comparison project^51^ (Supplementary Fig. 1). We additionally define four large regions encompassing most of Europe: Iberian Peninsula extending from −11 ° to 3.5 ° E and 34 ° to 44 ° N; continental central Europe encompassing the region between −5 ° to 25 ° E and 44 ° to 53 ° N, but excluding Great Britain; western Russia and eastern Europe extending from 29 ° to 60 ° E and 46 ° to 62 ° N; Scandinavia covering the region between 4.5 ° to 29 ° E and 56 ° to 71 ° N (Supplementary Fig. 1).

To evaluate the skill of inversions in capturing regional fluxes, we used the same synthetic runs from Jena inversion to evaluate: (i) the ability of the inversion to reproduce fluxes in large regions over Europe; (ii) the influence of the observational network. Despite the very coarse resolution, results (Supplementary Fig. 10) indicate that inversions do have moderate skill in distinguishing sub-regions within Europe. As expected from the better observational constrain, the skill increases with increasing number of stations. Nevertheless, even the s81 run (with few sites) is able to capture part of the regional differences, especially in eastern, central and western Europe. In south-western Europe (corresponding to the Iberian Peninsula), the inversion is not able to reproduce the fluxes, as expected from the very low observational constraint (only two sites in the complete MACCII set, located in the Pyrenees).

### NBP response to NAO–EA

All data fields were selected for the study region (Supplementary Fig. 1). Continental and regional NBP anomalies were calculated for each inversion and DGVM and evaluated for each of the four NAO–EA combinations (Fig. 2). In the case of MACC v11.2 inversion and JSBACH, the data sets do not encompass the whole period, therefore, the composites were calculated with fewer years. To partition annual NBP anomalies, continental and regional GPP and total respiration (autotrophic plus heterotrophic respiration components) were calculated at monthly scale, and compared with the corresponding NDVI anomalies (Supplementary Fig. 7).

For each composite, a one-sided analysis of variance (ANOVA) was performed to test the significance of the average anomaly value on the continental and regional scales (Supplementary Table 4), separately for inversions and DGVMs. Absolute NBP anomalies for each composite are easier to associate with NBP variations from year to year. However, it is worth assessing whether the inversions (or DGVMs) agree on the relative response, that is, the difference in NBP anomalies between two NAO–EA combinations. Therefore, a two-sided ANOVA was carried out to test the difference between NBP anomaly estimates for pairs of the four combinations (Supplementary Table 5), on the continental scale. Supplementary Tables 4 and 5 also include results of the ANOVA analyses for the neutral composite. To assess whether relationships hold for longer periods, the same analysis was extended for the period 1950–2012 using DGVMs (Supplementary Fig. 5).

Since the synthetic runs of Jena inversion present some dependence of absolute fluxes in the observational network, it is worth assessing how the number of sites may influence the results of NBP anomalies in response to NAO–EA variations. As the observational network has increased with time, Jena inversion is provided for different periods using an increasing number (always constant for each data set) of sites. Here we compare the other two inversions from Jena v3.6 that still encompass at least 20 years and use the same model but more sites: s85 (1985–2012, 19 sites), s90 (1990–2012, 25 sites). We also performed a run with the newer version of Jena inversion (v3.7) using the MACCII sites, at the same resolution as Jena s81 v3.6. The corresponding anomalies for each NAO–EA combination are presented in Supplementary Fig. 11 and show that, despite results depending on the size of the observational network, the response of continental NBP to the phases of NAO and EA for the data sets with more observations is consistent with the anomalies estimated by the two MACCII inversions and Jena s81 v3.6. In most cases, the inversion run using the MACCII sites is closer to the anomalies estimated by MACCII, highlighting the dependence of the anomalies on the observational constrains, but also the importance of other sources of uncertainty, for example, differences in the transport models.

## Additional information

**How to cite this article:** Bastos, A. *et al*. European land CO_2_ sink influenced by NAO and East-Atlantic Pattern coupling. *Nat. Commun.* 7:10315 doi: 10.1038/ncomms10315 (2016).

## Supplementary Material

Supplementary Information Supplementary Figures 1-11, Supplementary Tables 1-7 and Supplementary References

## Figures and Tables

**Figure 1:**
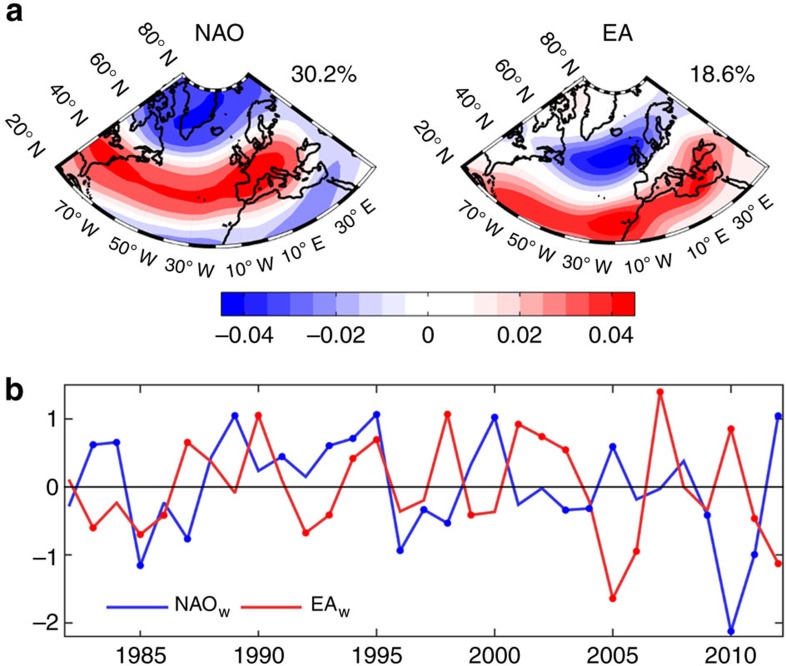
Main large-scale atmospheric circulation patterns in Europe. (**a**) Spatial patterns of the two first components of 500 mb geopotential height (from NCEP/DOEII Reanalysis) variability over the North-Atlantic: NAO and EA teleconnections. (**b**) Time series of the winter composites of NAO (blue line) and EA (red line) from NOAA; circle markers indicate the positive and negative phases of each mode, defined by the upper and lower tercile thresholds (Supplementary Table 3).
