# Supplementary material for: European land CO2 sink influenced by NAO and East-Atlantic Pattern coupling
Source: Nat Commun. 2016 Jan 18;7:10315. doi: 10.1038/ncomms10315 (PMC4735626; doi:10.1038/ncomms10315)
Supplement: Supplementary Information — Supplementary Figures 1-11, Supplementary Tables 1-7 and Supplementary References [file ncomms10315-s1.pdf]

Supplementary Figures

Supplementary Figure 1: Study region and observation sites from atmospheric inversions.

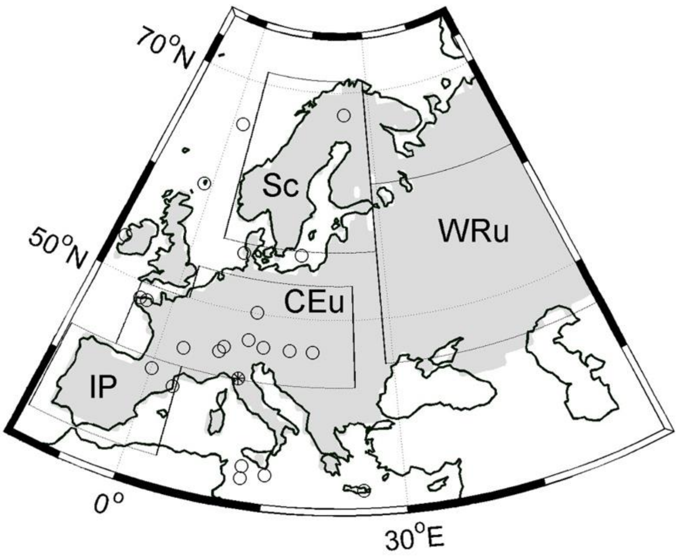

European area, as in TransCom, and the selected regions: IP – Iberian Peninsula, CEu - central Europe, WRu - western Russia / eastern Europe, and Sc - Scandinavia. The circles indicate MACCII, and asterisk Jena s81 v3.6 sites in Europe.

Supplementary Figure 2: Performance of models in assessing mean European NBP and corresponding variability.

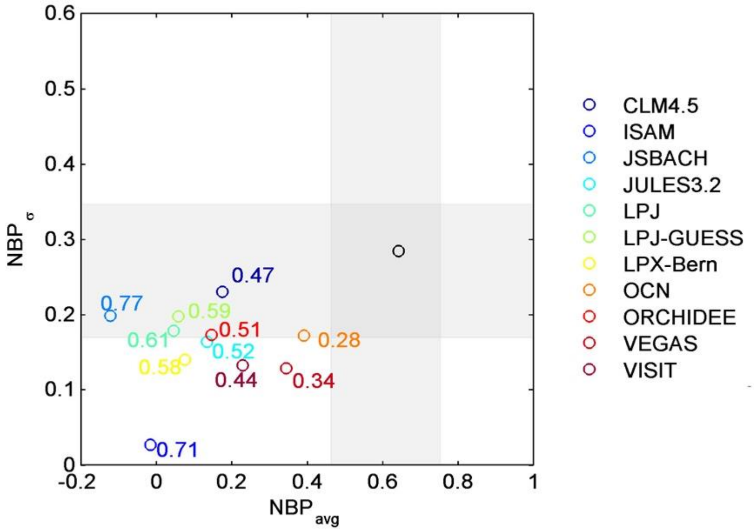

European sink evaluated by the mean of integrated NBP over Europe (*xx*) in PgC.yr^{-1}; standard-deviation of continental NBP (*yy*) is used as a measure of the variability of the sink. The black marker represents the median values for the inversions and the grey shades the spread between the three inversions. For each model, the distance to the inversion median is shown (numbers on the side of markers).

Supplementary Figure 3: Regional NBP variability.

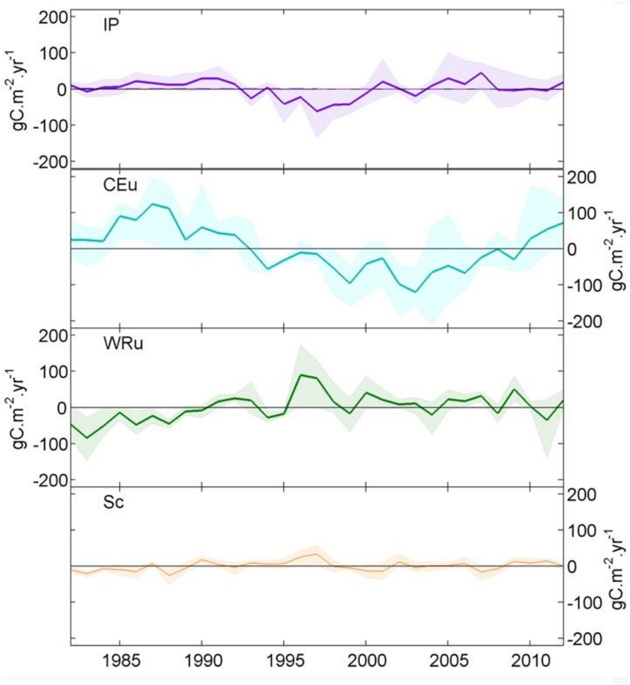

NBP anomalies from the inversion models in each of the four selected regions: average annual anomaly (bold lines) and the corresponding inversion spread (shaded areas).

Supplementary Figure 4: Analysis of the first component of inter-annual variability of European NBP.

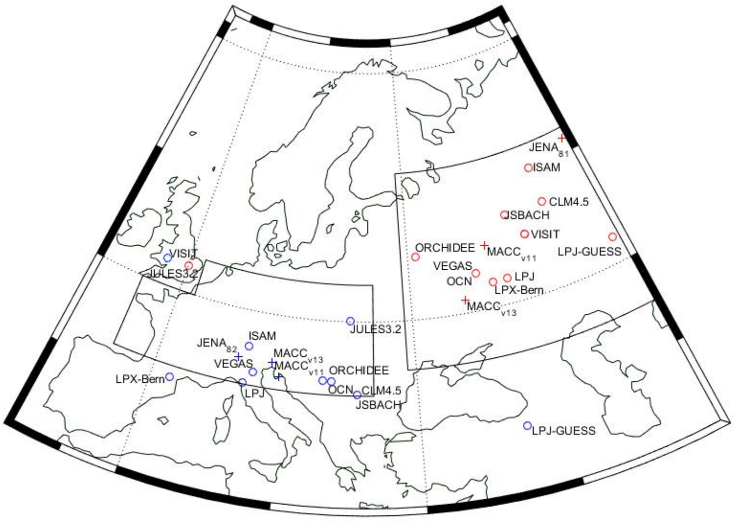

Location of the two centers (positive in red and negative in blue) of EOF1 for each inversion (+ markers) and DGVMs (circles). Inversions identify two centers located within the limits of CEu and WRu, as well as most of the DGVMs. VISIT and JULES3.2 have a one of their centers located over Great Britain, while LPJ-GUESS presents a center located over Turkey. For inversions the first principal component (PC1) explains from 32 to 77% of NBP variance, and for DGVMs from 11 to 42%. It must be noted that the model presenting the smaller variance explained by PC1 is LPJ-GUESS and the inversion in which PC1 explains 77% of the variance is JENA81, the one least constrained by observations and, therefore, more sensitive to atmospheric circulation variability.

Supplementary Figure 5: Extended assessment of European NBP response to NAO–EA phases.

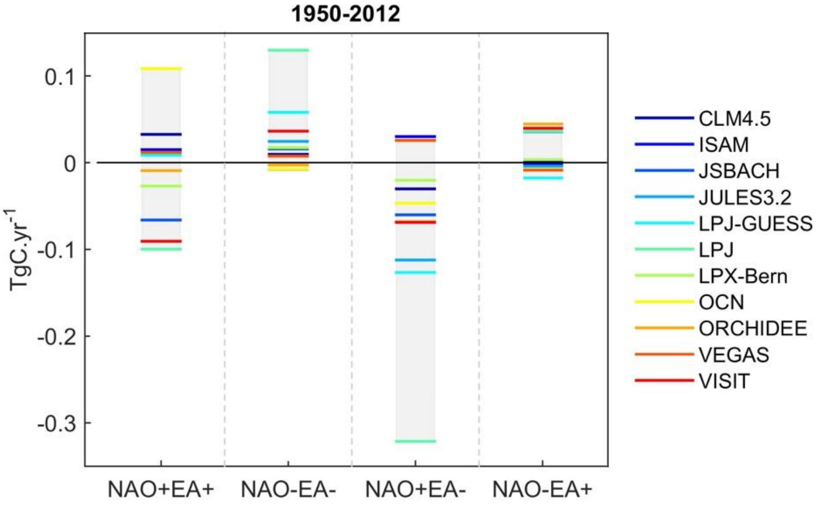

As in Figure 2b, but using the extended period from 1950 to 2012, only for DGVMs. Consistently with results for the shorter period, NAO[illegible]EA is the only composite presenting clearly positive NBP anomalies.

Supplementary Figure 6: Regional NBP response to NAO–EA phases.

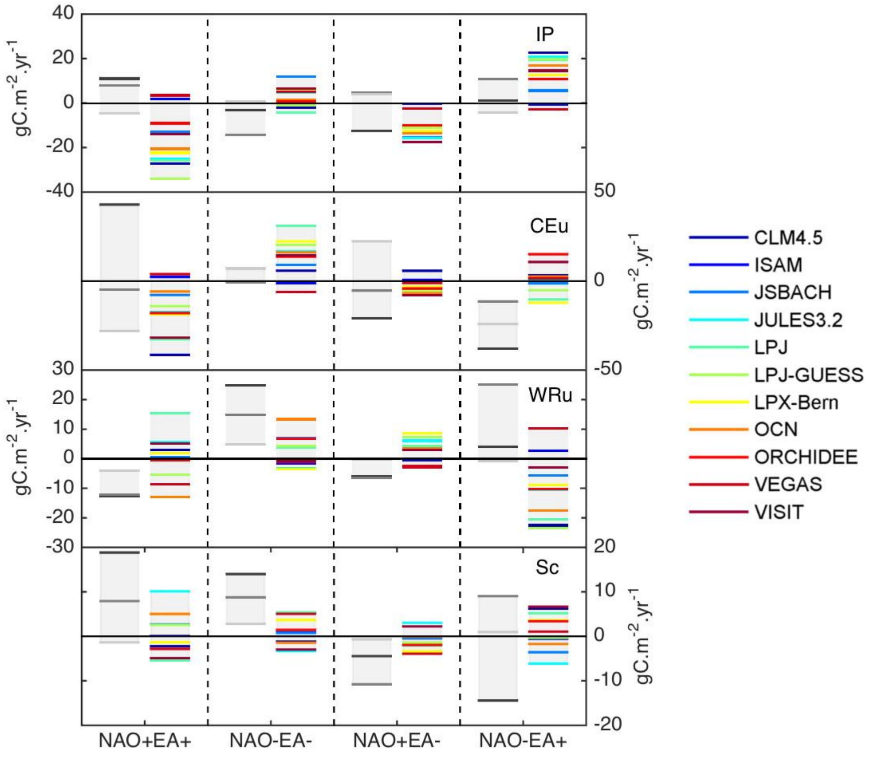

As in Fig. 2b but for average regional NBP estimated by inversions and DGVMs.

Supplementary Figure 7: Regional ecological response to NAO–EA phases.

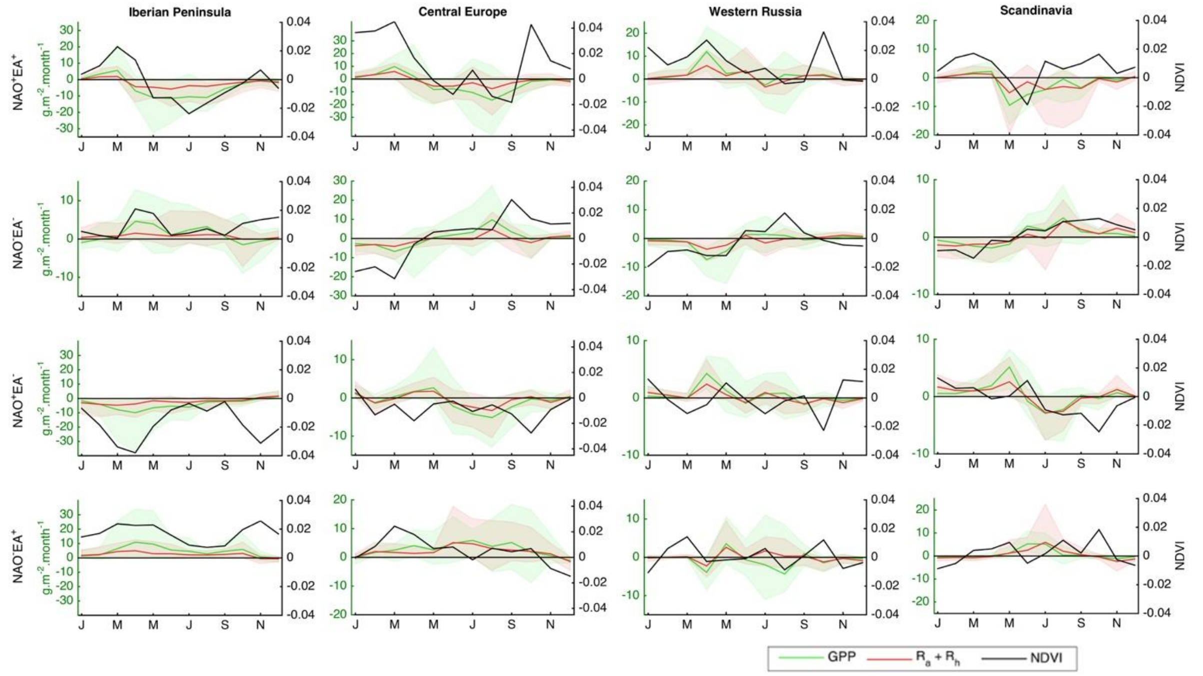

Seasonal anomalies of NDVI (black line, right *yy*-axis), GPP (green, left *yy*-axis) and aggregated autotrophic and heterotrophic respiration (red, left *yy*-axis) in each of the four selected regions. For GPP and respiration, bold line corresponds to the average of the eleven DGVMs and the shaded area indicates the model spread.

Supplementary Figure 8: Sensitivity of GPP from individual DGVMs to summer water availability during each of the four NAO–EA combinations.

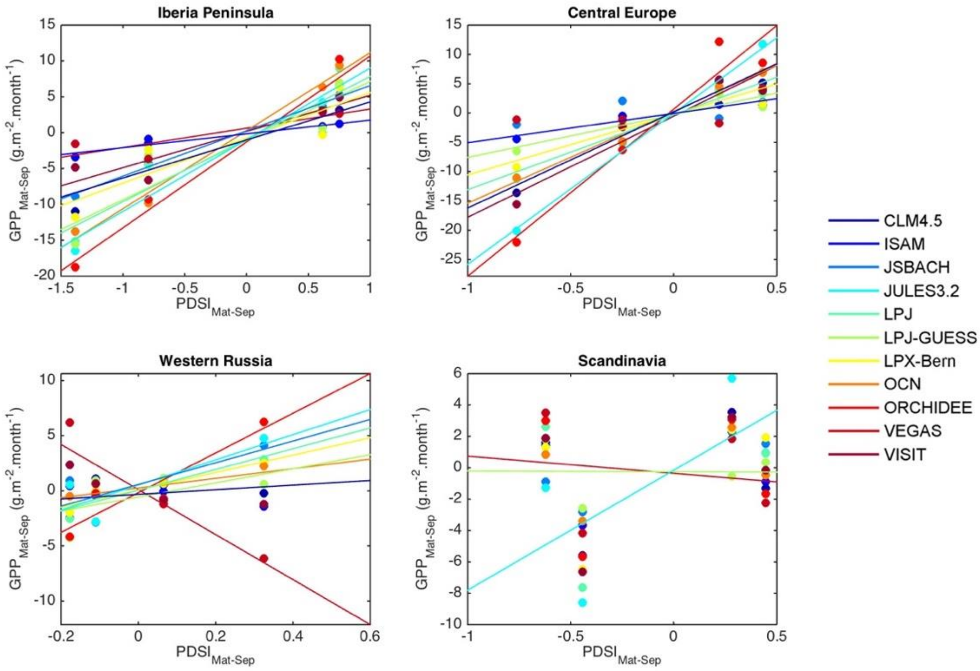

Average summer PDSI as in Tab. S6 (*xx*-axis) versus average GPP anomalies during May-Sep in each NAO–EA phase, estimated by the DGVMs. The regression lines are shown only for those models in which summer GPP is significantly influenced by soil water (Supplementary Table 7).

Supplementary Figure 9: Sensitivity of Jena inversion to the observation network.

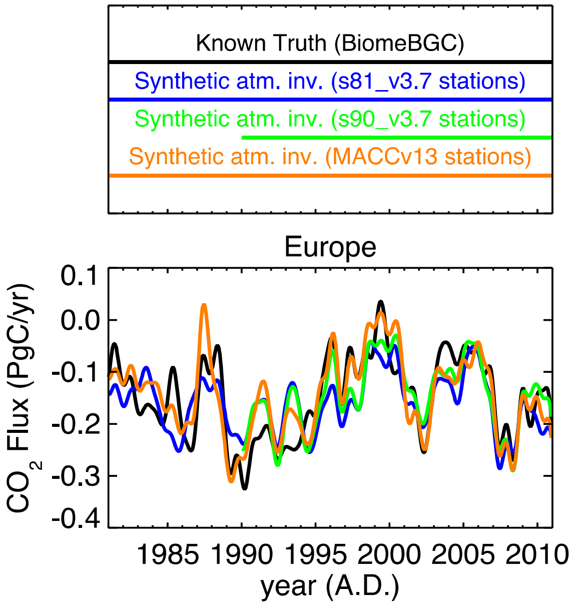

Synthetic runs of Jena inversion (1982-2010), performed by transporting NEE fields as simulated by the Biome-BGC DGVM in the atmosphere using the same transport model as in the Jena inversion (v3.7), though on coarser 10°x8° spatial resolution, at the same locations and times as the real data for 3 different station sets: as in Jena s81 (14 sites) and s90 (25 sites) as well as mostly the same sites used for MACCII inversion (see Methods).

Supplementary Figure 10: Evaluation of the regional performance and sensitivity of Jena inversion to the observation network.

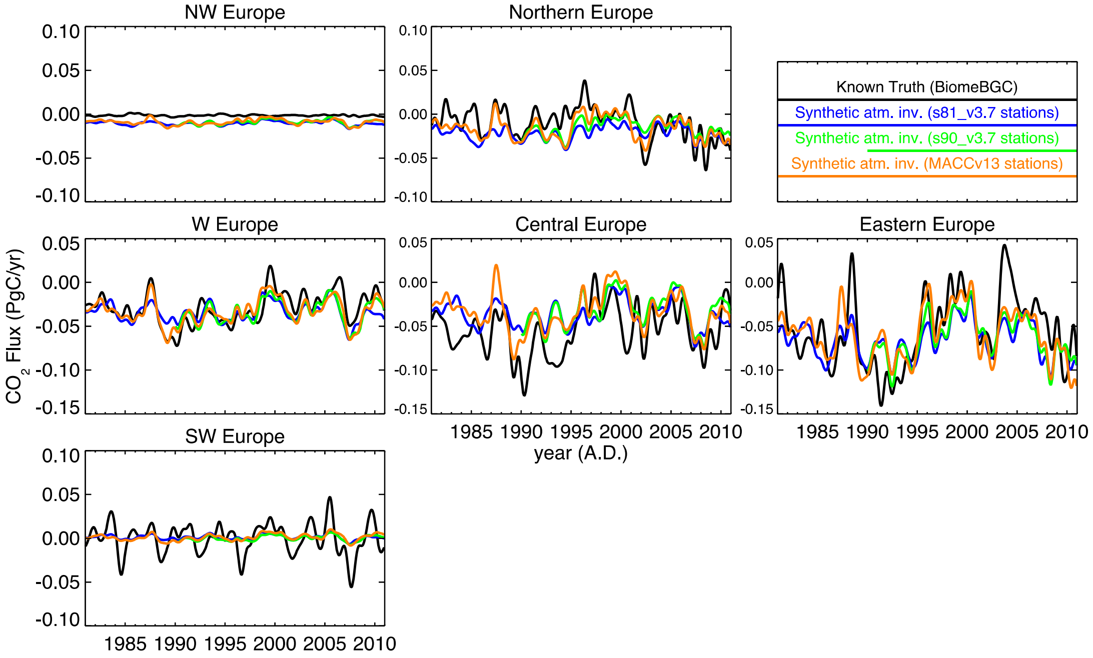

The same as in Supplementary Fig. 10, but for different regions within Europe.

Supplementary Figure 11: Influence of the observation sites number in NAO-EA response from Jena inversion.

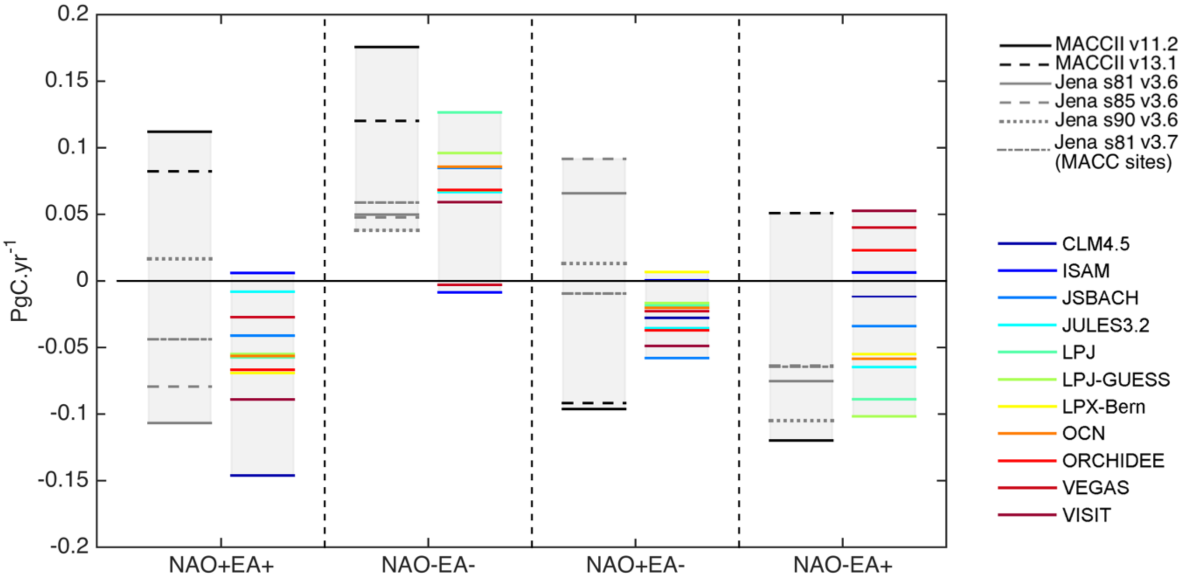

Continentally aggregated anomalies (as in Fig. 2b) for the inversions used in this work (MACCII in black, Jena s81 v3.6 in bold grey) and the other two inversions from Jena v3.6 that still encompass at least 20 years (dashed and dotted lines) and use the same model but more sites: s85 (1985-2012, 19 sites), s90 (1990-2012, 25 sites). See Methods for more details.

Supplementary Tables

Supplementary Table 1: Summary of the three atmospheric inversions used.

Inversion	Obs. sites	Period	Spatial resolution	Posterior uncertainty ($PgC.yr^{-1}$)	Reference
**MACC v11.2**	134	1982-2011	2.5^{o} x 3.75^{o}	0.50	[1]
**MACC v13.1**	134	1982-2012	1.9^{o} x 3.75^{o}	0.83	[1]
**Jena s81 v3.6**	14	1982-2012	4° x 5°	0.06	[2]

Supplementary Table 2: Summary of the eleven DGVMs used [3].

Model	Period	Spatial resolution	# PFTs	Vegetation	Fire	N-cycle	Reference
**CLM4.5**	1982-2012	1° x 1°	16	Imposed	Y	Y	[4]
**ISAM**	1982-2012	0.5^{o} x 0.5^{o}	9	Imposed	N	N	[5]
**JSBACH**	1982-2005	1.8^{o} x 1.8^{o}	13	Dynamic	Y	N	[6]
**JULES 3.2**	1982-2012	1.88^{o}x 1.25^{o}	5	Dynamic	N	N	[7]
**LPJ**	1982-2012	0.5^{o} x 0.5^{o}	11	Dynamic	Y	N	[8]
**LPJ-GUESS**	1982-2012	0.5^{o} x 0.5^{o}	11	Dynamic	Y	N	[8]
**LPX-Bern**	1982-2012	1° x 1°	11	Dynamic	Y	N	[9]
**OCN**	1982-2012	1° x 1°	12	Imposed	Y	Y	[10]
**ORCHIDEE**	1982-2012	2° x 2°	12	Imposed	Y	N	[11]
**VEGAS**	1982-2012	0.5^{o} x 0.5^{o}	4	Dynamic	N	N	[12]
**VISIT**	1982-2012	0.5^{o} x 0.5^{o}	5	Imposed	N	Y	[13]

The period shown is the one used in the main analysis. For Figure S5, all records start in 1950.

Supplementary Table 3: Years corresponding to the four NAO–EA composites.

In-phase		Anti-phase		
NAO+EA+	NAO-EA-	NAO+EA-	NAO-EA+	Neutral
1990	1985	1983	1987	1982
1991	1986	1984	1998	1988
1994	1996	1989	2001	2008
1995	1997	1992	2002	-
-	2004	1993	2003	-
-	2006	1999	2007	-
-	2009	2000	2010	-
-	2011	2005	-	-
-	-	2012	-	-

Combined phases correspond to years in which one of the indices (NAO or EA) ≥ upper tercile (+) or ≤ lower tercile (-), as the other has the corresponding, or inverse, sign. The neutral set corresponds to the remainder of the years in which neither NAO nor EA were in a strong phase.

Supplementary Table 4: Statistical analysis of NBP anomalies at the continental and regional scales.

	Inversions				
	NAO+EA+	NAO-EA-	NAO+EA-	NAO-EA+	Neutral
Europe	0.36+	**0.04+**	0.26-	0.22-	0.15-
IP	0.21+	0.17-	0.42-	0.31+	0.32+
CEu	0.44-	0.12+	0.46-	**0.04-**	**0.07+**
WRu	**0.04-**	**0.06+**	**0.08-**	0.18+	**0.08-**
Sc	0.14+	**0.06+**	0.11-	0.43+	**0.05-**
	DGVMs				
	NAO+EA+	NAO-EA-	NAO+EA-	NAO-EA+	Neutral
Europe	**<<0.01-**	**<<0.01+**	**<<0.01-**	**0.06-**	**0.02+**
IP	**<<0.01-**	**0.04+**	**<<0.01-**	**<<0.01+**	**<<0.01+**
CEu	**<<0.01-**	**<<0.01+**	**0.01-**	0.44+	0.29+
WRu	0.45+	**0.05+**	**0.06+**	**0.01-**	**0.02+**
Sc	0.48+	0.17+	**0.07-**	0.17+	**0.10**-

Significance (*p-values*) of results from the ANOVA performed on the sets of average regional anomalies during NAO-EA phases for inversions and DGVMs, as well as for the neutral composite, for comparison. The + and - signs indicate the sign of the corresponding anomaly. Values significant at the 10% level are highlighted in bold. Results of anomaly sign for the neutral set of years are generally not consistent, excepting for Sc, and may relate to other processes.

Supplementary Table 5: Statistical analysis of the difference in NBP anomalies between pairs of the four NAO–EA combinations.

		Inversions				
		NAO+EA+	NAO-EA-	NAO+EA-	NAO-EA+	Neutral
DGVMs	NAO+EA+	-	0.17+	0.23-	0.21-	0.13-
	NAO-EA-	**<<0.01+**	-	**0.04-**	**0.03-**	**0.05-**
	NAO+EA-	**0.02+**	**<<0.01-**	-	0.46-	0.26-
	NAO-EA+	**0.08+**	**<<0.01-**	0.47-	-	0.28-
	Neutral	**<<0.01+**	0.11-	**<<0.01+**	**<<0.01+**	-

Significance (*p-values*) of results from the two-sided ANOVA for two distributions with unequal variance, performed on the sets of average NBP anomalies on the continental scale (Figure 2) for inversions (entries above the main diagonal) and DGVMs (entries below the main diagonal). Values significant at the 10% level are highlighted in bold. The + and - signs indicate the sign of the corresponding difference (for inversions the reference are the combinations on the top row, for DGVMs, the combinations on the left column) e.g. NBP anomalies for NAO-EA- are bigger than anomalies for NAO+EA+: *p-value* of inversions 0.17 and DGVMs <<0.01.

Supplementary Table 6: Regional drought conditions for the four NAO–EA phases.

		NAO+EA+	NAO-EA-	NAO+EA-	NAO-EA+
IP	Jan-Apr	-0.54	0.94	-1.32	0.90
	May-Sep	-1.39	0.62	-0.79	0.75
CEu	Jan-Apr	-0.63	-0.03	-0.18	0.11
	May-Sep	-0.76	0.22	-0.25	0.43
WRu	Jan-Apr	0.65	-0.43	0.08	0.09
	May-Sep	0.32	-0.11	0.07	-0.18
Sc	Jan-Apr	-0.14	-0.92	0.83	-0.22
	May-Sep	-0.44	-0.62	0.45	0.28

Mean values of the self-calibrated PDSI index for each of the four NAO–EA composites. Values presented correspond to the departure of the regional mean, since PDSI presented regional dependence (negative for IP and CEu and positive for WRu and Sc). Negative values indicate dry relative conditions and positive correspond to wet relative conditions.

Supplementary Table 7: Sensitivity of regional GPP to drought in individual DGVMs.

	Jan-Apr		May-Sep							
	IP		IP		CEu		WRu		Sc	
	Slope	R^2	Slope	R^2	Slope	R^2	Slope	R^2	Slope	R^2
CLM4.5	**1.5**	**0.17**	**4.9**	**0.54**	**10.3**	**0.55**	**7.6**	**0.43**	0.1	0.00
ISAM	0.1	0.01	**1.0**	**0.32**	1.1	0.10	0.5	0.02	-1.1	0.05
JSBACH	1.3	0.07	**5.1**	**0.59**	-2.1	0.08	3.3	0.13	-1.4	0.03
JULES 3.2	**5.3**	**0.60**	**9.8**	**0.69**	**17.2**	**0.59**	**14.3**	**0.57**	**4.5**	**0.16**
LPJ	0.6	0.01	**7.4**	**0.48**	**6.3**	**0.34**	**9.7**	**0.53**	-0.8	0.00
LPJ-GUESS	-0.1	0.00	**7.4**	**0.52**	**4.8**	**0.35**	**8.1**	**0.54**	**-2.8**	**0.16**
LPX-Bern	0.2	0.00	**5.3**	**0.41**	**5.3**	**0.30**	**7.2**	**0.35**	0.4	0.00
OCN	**1.6**	**0.17**	**7.9**	**0.73**	**9.3**	**0.37**	**11.5**	**0.51**	-1.9	0.06
ORCHIDEE	**6.0**	**0.32**	**11.6**	**0.60**	**14.8**	**0.52**	**13.7**	**0.64**	-2.0	0.04
VEGAS	0.3	0.01	1.1	0.12	-2.7	0.05	**-4.7**	**0.14**	**-4.1**	**0.19**
VISIT	2.3	0.36	**4.1**	**0.63**	**5.5**	**0.26**	-0.3	0.00	-2.2	0.04

Slope (in $gC.m^{-2}.month^{-1}.PDSIunit^{-1}$) and R^2 values of the linear model fit on regional GPP (in Jan-Apr in IP and May-Sep in all regions) and the corresponding self-calibrated PDSI values. Significant values at 5% level are highlighted in bold.

Supplementary References

[1] Chevallier, F. et al. CO_2 surface fluxes at grid point scale estimated from a global 21-year reanalysis of atmospheric measurements. J. Geophys. Res., 115, D21307 (2010).

[2] Estimating CO2 sources and sinks from atmospheric mixing ratio measurements using a global inversion of atmospheric transport. Technical Report 6, Max Planck Institute for Biogeochemistry (2005).

[3] Sitch, S. et al. Trends and drivers of regional sources and sinks of carbon dioxide over the past two decades. Biogeosciences Discussions, 10, 20113–20177 (2013).

[4] Oleson, K. et al. Technical description of version 4.5 of the Community Land Model (CLM). Technical Note NCAR/TN-503+STR, NCAR (2013).

[5] Barman, R., Jain, A. K. & Liang, M. Climate-driven uncertainties in modeling terrestrial Gross Primary Production: a site level to global-scale analysis. Global Change Biology, 20, 1394–1411 (2014).

[6] Raddatz, T. et al. Will the tropical land biosphere dominate the climate–carbon cycle feedback during the twenty-first century? Clim. Dyn., 29, 565–574 (2007).

[7] Cox, P. M. Description of the TRIFFID Dynamic Global Vegetation Model Technical Note 24, Hadley Centre, United Kingdom Meteorological Office, Bracknell, UK (2001).

[8] Sitch, S. et al. Evaluation of ecosystem dynamics, plant geography and terrestrial carbon cycling in the LPJ Dynamic Global Vegetation Model, Global Change Biology, 9, 161–185 (2003).

[9] Joos, F. et al. Global warming feedbacks on terrestrial carbon uptake under the Intergovernmental Panel on Climate Change (IPCC) emission scenarios. Global Biogeochem. Cycles, 15, 891–907 (2001).

[10] Zaehle, S. et al. Carbon and nitrogen cycle dynamics in the O-CN land surface model: 2. Role of the nitrogen cycle in the historical terrestrial carbon balance. Global Biogeochem. Cycles 24, GB1006 (2010).

[11] Krinner, G. et al. A dynamic global vegetation model for studies of the coupled atmosphere-biosphere system. Global Biogeochem. Cycles, 19, GB1015 (2005).

[12] Zeng, N., Mariotti, A. & Wetzel, P. Terrestrial mechanisms of interannual CO_2 variability. Global Biogeochem. Cycles, 19, GB1016 (2005).

[13] Ito, A. Changing ecophysiological processes and carbon budget in east Asian ecosystems under near-future changes in climate: implications for long-term monitoring from a process-based model, Journal of Plant Research, 123, 577–588 (2010).
